# Right Coronary Artery Originated from the Left Anterior Descending Artery in a Patient with Congenital Pulmonary Valvular Stenosis

**DOI:** 10.1155/2013/413961

**Published:** 2013-02-06

**Authors:** Yusuf Hoşoğlu, Cihan Örem, Oğuzhan Ekrem Turan, Mustafa Öztürk, Ömer Gedikli, Ayşe Hoşoğlu, Mürsel Şahin

**Affiliations:** ^1^Department of Cardiology, Faculty of Medicine, Karadeniz Technical University, Trabzon, Turkey; ^2^Kardiyoloji ABD, KTÜ Tıp Fakültesi, 61080 Trabzon, Turkey

## Abstract

The single coronary artery, anomalous origin of the right coronary artery from the left anterior descending artery, is a benign and very rare coronary artery anomaly. We firstly present a case with this type of single coronary artery and congenital pulmonary valvular stenosis with large poststenotic dilatation.

## 1. Introduction

The term single coronary artery defines coronary arteries originating from a single coronary ostium in the aorta. A type of single coronary artery, right coronary artery (RCA) originating from the left anterior descending artery (LAD), is an extremely rare coronary artery abnormality (incidence 0.024%) [1]. Most of the these reported cases have been in structurally normal hearts [2].

Aneurysms of the pulmonary trunk are rare lesions. These aneurysms are usually associated with cardiac malformations resulting in pulmonary hypertension and pulmonary stenosis [3]. Other causes can be idiopathic or associated with several processes including infections, traumatisms, or diseases affecting collagenous tissue [4–7]. Poststenotic dilatation secondary to pulmonary stenosis is quite common, but it rarely progresses to an aneurysm.

In this paper, we firstly reported a patient with a single coronary artery, in whom the RCA originated from the proximal of the LAD with congenital pulmonary valvular stenosis and poststenotic large pulmonary artery (PA) dilatation.

## 2. Case Report

A 62-year-old woman was referred to us with suspicion of pulmonary hypertension, due to PA dilatation, in Department of Chest Diseases. Her complaint was shortness of breath during last 3 months. She had been told that she had a congenital heart disease. In physical examination, 3/6 systolic ejection murmur, best heard at left side, was observed. Other physical findings were normal. Electrocardiography (ECG) was in sinus rhythm. No ischemic ST-T changes were observed. There was a widened mediastinum in chest X-ray.

In transthoracic echocardiography, left ventricular measurements and functions were normal. Right ventricle was nearly dilated. There was main pulmonary artery dilatation (46 mm) and moderate pulmonary valvular stenosis (maximum gradient 46 mmHg, mean gradient 30 mmHg). Systolic pulmonary artery pressure (PAP) could not be measured with Doppler echocardiography because of pulmonary stenosis. Echocardiographic images could not be shown as figure due to poor echocardiographic imaging. To evaluate any other congenital anomalies and measure PAP and gradients, coronary angiography and right-sided cardiac catheterization were planned.

**Figure 1:**
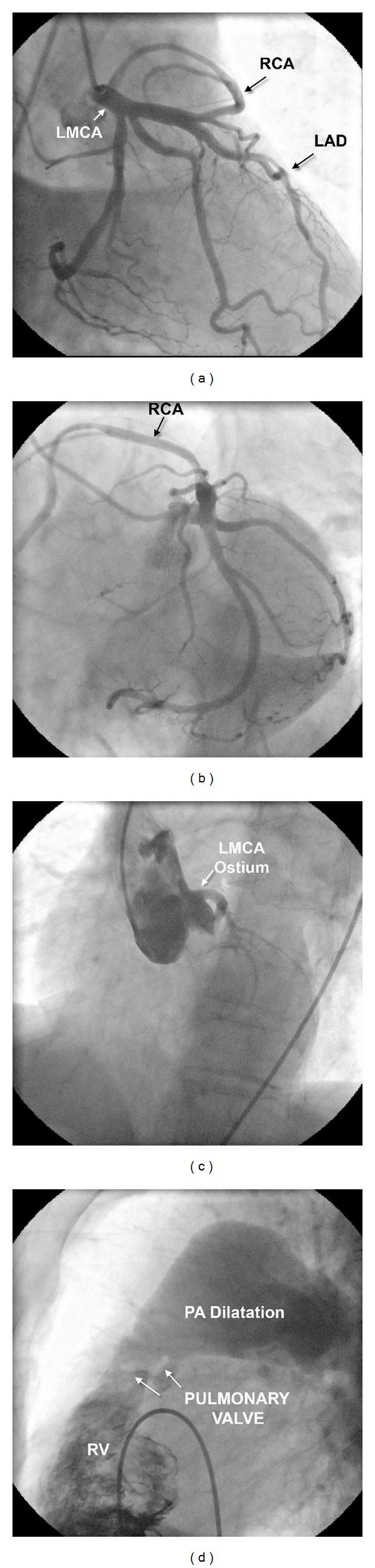
(a) Right anterior oblique view with caudal angulation. The RCA originates from the proximal portion of the LAD and travels to the right ventricle area. (b) Left anterior oblique view. The course of the anomalous RCA after its origin from the LAD. (c) Left anterior oblique view. Aortic root angiography revealed no coronary artery arising from the right sinus of Valsalva. (d) RV and PA angiogram showing calcific pulmonic valve (arrow) with poststenotic dilatation of the pulmonary arteries. RCA: right coronary artery. LAD: left anterior descending artery. RV: right ventricle. PA: pulmonary artery.

During coronary angiography, left coronary ostium was located normally, and selective left coronary angiography demonstrated the normal left main, LAD, and dominant left circumflex (Cx) arteries. An anomalous RCA, as a separate branch, arose from the proximal of LAD after the first septal perforator and coursed anterior to the right ventricular outflow tract to gain the right atrioventricular sulcus (Figures 1(a) and 1(b)). The RCA could not be cannulated in the right sinus of Valsalva. Aortagraphy showed no coronary ostium originating from the right sinus of Valsalva (Figure 1(c)). No significant stenosis could be observed in any of the coronary arteries. Right ventricular angiograms revealed evidence of doming of the calcified pulmonic valve with a large poststenotic dilatation involving the main pulmonary artery and its branches (Figure 1(d)). Systolic PAP pressure was 14 mmHg, and systolic right ventricular pressure was 47 mmHg. Peak-to-peak pulmonary gradient was observed 33 mmHg. Qp/Qs was calculated as 1,1.

**Figure 2:**
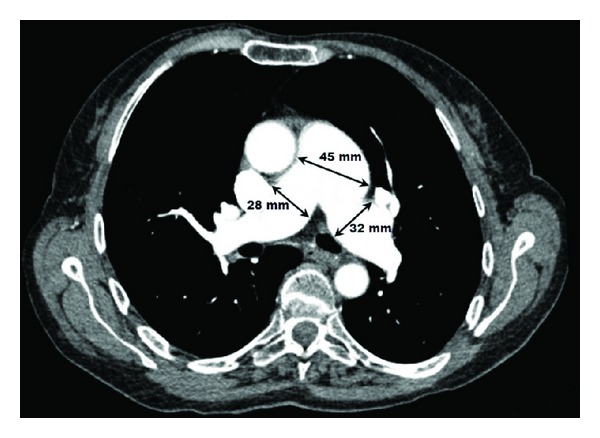
Computerized tomography of the chest showing large PA dilatation involving the main PA and its branches. PA: pulmonary artery.

In order to evaluate PA, pulmonary computed tomographic angiography was planned. Diameters of main PA, right and left PA, were 45 mm, 28 mm, and 32 mm, respectively (Figure 2). There was no dissection or another anomalies. Multislice coronary computed tomographic (cCT) angiogram was planned to evaluate course of RCA. Mechanical compression of the anomalous RCA between the aorta and pulmonary artery might be related ischemia. But, it could not be performed due to technical insufficiency in our hospital. Therefore, exercise stress test was planned to determine presence of ischemia. During the stress test, ischemic signs were not observed.

Patient was evaluated by cardiothoracic surgeons for surgical treatment. They suggested conservative management and followup because of low PAP pressures and moderate pulmonary stenosis. It was suggested orally a *β*-blocker (50 mg of metoprolol) and aspirin (100 mg) treatment due to minimal coronary heart disease. During 6 months followup, no clinical and hemodynamic changes were observed in the patient.

## 3. Discussion

Anomalous origin of RCA from LAD has rarely been reported in the literature. We are able to find only 37 published cases with this specific coronary anomaly [8, 9]. Most of the these reported cases have been in structurally normal hearts, and there are only two cases in association with Tetralogy of Fallot [10, 11]. Therefore, according to our knowledge, the current case is the first report with RCA originating from the LAD artery and also with congenital pulmonary valvular stenosis and large poststenotic dilatation.

This type of single coronary artery is a usually benign anomaly, and clinical significance is associated with the course of the anomalous originated RCA [12]. Mechanical compression of the anomalous RCA between the aorta and pulmonary root might be related ischemia in the absence of coronary artery disease. It was suggested that multislice cCT better delineates the anatomic course of anomalous RCA and whether it passes between the aortic and pulmonary trunks [9, 13]. As mentioned above, cCT could not be performed in our case. Although there was a large PA dilatation, coronary ischemia was not observed in our patient by clinical evaluating and using rest and exercise ECG. These findings can support that anomalous originated RCA did not course between aorta and PA. The vast majority of previous reports [2] have described a single anomalous vessel originated after the first septal perforator of the LAD, which courses anterior to the right ventricular outflow tract to reach territory normally served by the RCA, as seen in present patient.

The distinction between secondary poststenotic dilatation of the PA and definite aneurysm is controversial. Previously, histologic evidence of arterial wall damage was required for definite diagnosis of aneurysm. Now, since it has been demonstrated that the walls of the PA aneurysm may be histologically normal, a size of ≥5 cm in diameter is accepted as an aneurysm [3]. Most of the autopsy data reveals poststenotic dilatation in the 3.0–3.5 cm diameter range [3]. In current case, there was quite large a PA dilatation, but it was not accepted as an aneurysm because it was lower than 5 cm.

The natural history of large PA dilatation or aneurysms is largely unknown at present, and there are no definite guidelines for management. Even if it is unclear, surgery is indicated, if the risk of rupture exists [14]. Patients with <6 cm aneurysms, low PAP, absence of congenital or acquired significant right-left shunt, or not associated with collagenopathies are considered at low risk, and conservative management is preferred, as in our patient [15, 16].

In this report, we presented a patient with a rare and incidentally discovered coronary anomaly when we investigated etiology of PA dilatation. We concluded that it is important to recognise the presence of coronary anomalies when structural heart disease was evaluated.
